# *Patrinia scabiosaefolia* L. Modulates the Intestinal Microecology to Treat DSS-Induced Ulcerative Colitis: UHPLC-OE-MS/MS, Network Pharmacology, and Experimental Validation

**DOI:** 10.3390/foods14071145

**Published:** 2025-03-25

**Authors:** Longfei Zhang, Xiaoxiao Liu, Mingze Xu, Xinyi Cheng, Ning Li, Haiyan Xu, Yining Feng, Tianzhu Guan, Lixia Xiao

**Affiliations:** 1 College of Food Science and Technology, Yangzhou University, Yangzhou 225000, China; zlfjssz@126.com (L.Z.); lxxyzu@163.com (X.L.); xumingze45@gmail.com (M.X.); 13327831705@163.com (X.C.); ningliyzu@163.com (N.L.); 19852318085@163.com (H.X.); mx120241326@stu.yzu.edu.cn (Y.F.); guantz@yzu.edu.cn (T.G.); 2 Jiangsu Provincial Key Laboratory for Probotics and Dairy Deep Processing, Yangzhou 225000, China

**Keywords:** *Patrinia scabiosaefolia*, ulcerative colitis, UHPLC-OE-MS/MS, intestinal microecology

## Abstract

*Patrinia scabiosaefolia* L. (*P. scabiosaefolia*), a traditional food and medicinal plant, is used to treat internal inflammation. This study investigated the mechanisms by which *P. scabiosaefolia* improves ulcerative colitis (UC) via combined UHPLC-OE-MS/MS, network pharmacology, molecular docking, and animal experiments. A total of 72 compounds were detected in the *P. scabiosaefolia* extraction, with 15 key components (ranking by degree value) selected for further analysis. GO enrichment analysis suggested that PS may alleviate UC-related renal dysfunction by modulating immune responses, inflammation, and cell signaling pathways. Based on protein–protein interaction results, five core targets of *P. scabiosaefolia* in UC (ranking by degree value) were identified, and molecular docking revealed strong binding free affinity (<−7 kcal/mol) of active components (Vulgarin and 4-(Diphenylphosphino)benzoic acid) with TNF, AKT1, CASP3, BCL2, and MMP9. In animal experiments, *P. scabiosaefolia*-treated mice showed significant reductions in IL-6, TNF-α, LPS, and D-Lactate levels (*p* < 0.05); improved colon histopathological damage; and significantly increased the mRNA expression of tight junction proteins (ZO-1, Claudin, OCC) in colon tissue (*p* < 0.05). Furthermore, *P. scabiosaefolia*-treated mice exhibited a significant increase in beneficial gut bacteria (*Enterococcus* and *Lactobacillus*) (*p* < 0.05), effectively restoring the gut imbalance caused by DSS. In conclusion, *P. scabiosaefolia* can treat UC through the modulation of the intestinal microecology.

## 1. Introduction

Ulcerative colitis (UC) is the most prevalent form of inflammatory bowel disease (IBD), marked by a challenging prognosis, recurrent flare-ups, and a risk of developing colitis-associated cancer [1]. Epidemiological data indicate that by 2023, the global number of UC cases is expected to reach 5 million, with an increasing incidence rate [2]. UC is driven by an abnormal immune response targeting the gut microbiome, resulting from the complex interaction of genetic susceptibility and environmental factors [3]. Classic symptoms of UC include rectal bleeding, increased stool frequency, and urgency of defecation [4]. The pathophysiology of UC involves key mechanisms such as inflammation, cell adhesion molecules, and alterations in the gut microbiota [5]. While treatments like biologics, 5-aminosalicylic acid, immunosuppressants, and corticosteroids can provide partial symptom relief, their use remains limited by various factors [6]. Consequently, there is an increasing focus on identifying new, effective natural therapies.

*Patrinia scabiosaefolia* L. (*P. scabiosaefolia*), belonging to the Valerianaceae family, is mainly distributed in East Asia, Central Asia, and the northwestern regions of North America. The entire *P. scabiosaefolia* are used as vegetables for soup in East Asia, while the plant has traditionally been utilized to clear heat, detoxify, and moisten the intestines [7,8]. Previous studies have shown that *P. scabiosaefolia* extract exhibits strong therapeutic effects in treating appendicitis, enteritis, and hepatitis [9]. Liu et al. found that the compounds patrinoside and patrinoside A, present in *P. scabiosaefolia*, have the capacity to suppress the expression of pro-inflammatory mediators such as IL-6, as well as chemokines including MCP-1 and MIP-1α within the RAW264.7 cellular framework. This suppression leads to the downregulation of both NF-κB and MAPK signaling cascades, contributing to the amelioration of oxidative stress within the organism [10]. Additionally, *P. scabiosaefolia* effectively inhibits the growth of the harmful bacterium *Staphylococcus epidermidis* [9]. Recent studies have also shown that *P. scabiosaefolia* possesses the ability to modulate the gut microbiota [11]. This factor is pivotal in the pathological mechanisms underlying UC.

Intestinal microecology refers to the complex and dynamic ecosystem within the gastrointestinal tract, consisting of the gut microbiota, the intestinal structure, and the contents within the intestine [12]. The intestinal microecology is a dynamic system that not only contributes to nutrient absorption but also regulates immune responses and protects against infections. Imbalances in this ecosystem can result in gastrointestinal disorders, highlighting the importance of maintaining a healthy gut microbiome and intestinal environment for overall health. Several studies have shown that a reduction in beneficial bacteria and an increase in harmful bacteria contribute to the worsening of UC symptoms [13]. A recent study found that tight junctions (TJs) are a key component of the intestinal barrier, maintaining the selective permeability of the intestinal epithelial barrier. TJs prevent the entry of pathogenic antigens from the gut lumen into the mucosal layer, which could lead to intestinal, and even systemic, inflammation and immune responses [14]. Furthermore, the disruption of the gut microbiota increases the concentration of lipopolysaccharides (LPSs) in the intestine, which activates signaling pathways such as TLR4/NF-κB and MAPK, further intensifying the inflammatory response in the intestinal tissues [10].

There has been increasing interest in the potential of food-derived bioactive components to modulate the gut microbiota as a strategy for treating UC. However, the role of *P. scabiosaefolia* in this regard remains incompletely understood. Therefore, the study aims to identify the key components of *P. scabiosaefolia* extract using UHPLC-QE-MS/MS and to predict the potential mechanisms underlying *P. scabiosaefolia*’s therapeutic effects on UC through network pharmacology and molecular docking. Additionally, a DSS-induced UC mouse model was established, and the therapeutic effects of *P. scabiosaefolia* on DSS-induced UC were thoroughly assessed by monitoring body weight changes, inflammation levels, histopathological alterations, and the balance of the colon microbiota. The findings from this study will provide a solid theoretical foundation for developing *P. scabiosaefolia* as a functional food or therapeutic agent.

## 2. Materials and Methods

### 2.1. Extraction of P. scabiosaefolia

*P. scabiosaefolia* was purchased from Huaishuntang Pharmaceutical Co., Ltd. (Bozhou, China). *P. scabiosaefolia* was dried and then subjected to high-speed pulverization. The preparation of *P. scabiosaefolia* via ultrasonic-assisted extraction method was conducted according to the previous method with minor modifications. Then, an ultrasonic-assisted extraction method was employed with a material–water ratio of 1:10 (*w*/*v*) at 65 °C and an ultrasonic treatment duration of 175 min. After the extraction, the mixture was centrifuged at 3000 r/min for 10 min.

### 2.2. UHPLC-OE-MS/MS

The *P. scabiosaefolia* composition was analyzed using a Dionex 3000 Ultimate UHPLC and Q-Exactive Orbitrap HRMS/MS (Thermo Fisher, Waltham, MA, USA). An Acquity UPLC BEN C18 Column (4.6 × 100 mm, 1.8 μm) was operated at 40 °C. An MS analysis was carried out in the positive and negative ion modes.

### 2.3. Network Pharmacology

Based on the UHPLC-OE-MS/MS abovementioned, the study selected substances with MS2 score > 2.0 and relative peak area > 0.5% for the subsequent target prediction. The corresponding potential targets were predicted by using Swiss Target Prediction (http://www.swisstargetprediction.ch/, accessed on 10 December 2024). Potential protein targets associated with UC were identified by analyzing two comprehensive databases: CTD (available via http://ctdbase.org/, accessed on 15 December 2024) and OMIM (accessible via https://omim.org/, accessed on 15 December 2024). The overlapping targets between *P. scabiosaefolia* and UC were selected as the definitive protein targets. A functional enrichment analysis of these targets was conducted using the DAVID platform (https://david.ncifcrf.gov/tools.jsp, accessed on 20 December 2024), followed by the visualization of the results. The interaction network of PS-UC key targets was constructed using Cytoscape 3.7.1.

### 2.4. Molecular Docking

The top five compounds with the highest degree values from the active ingredient–target network of *P. scabiosaefolia* were chosen as ligands. Similarly, the top five targets with the highest degree values were selected from the protein–protein interaction (PPI) network of shared drug-disease targets. These identified targets were subsequently queried in the Protein Data Bank database (accessible via https://www.rcsb.org/, accessed on 25 December 2024). The AutoDock Vina was used to perform molecular docking of the receptor protein with the drug ligand, the results were saved, and the binding affinity values were recorded and analyzed. The 10 binding force compounds were imported into PyMOL 3.0.3 for further visualization.

### 2.5. Animal Experiment

#### 2.5.1. Experiment Design

Male C57BL/6 mice with a body weight of 18 ± 2 g, 6–8 weeks old were purchased from Yangzhou University (Yangzhou, China, Permission Number: SCXK2016-0006). The experiment was ethically approved by the Institutional Animal Ethics Committee of the School of Yangzhou University (Jiangsu, China). After adaptation feeding, the mice were randomly divided into the control group, DSS group, P-control group, and PS group (*n* = 7). The mice in the DSS group, P-control group, and PS group were administered drinking water containing 5% (*w*/*v*) DSS for 7 days. The control group was given regular drinking water without DSS [15]. Starting on day 8, the P-control group was gavaged with commercially available Rou Suan Ku Shen Jian (RSKSJ) granules (1.0 mL/20 g per day) for 3 weeks, while the PS group received *P. scabiosaefolia* extract via gavage (1.0 mL/20 g per day). The equivalent dose of the drug was calculated based on the body surface area of both humans and animals, with the daily dose for mice being approximately 6.25 times that of an adult human. The mice in the control and DSS groups were gavaged with an equal volume of saline. The mice were housed in a clean animal room with a temperature of 24–26 °C, relative humidity of 50–60%, and a 12 h light–dark cycle. Feed was supplied by Jiangsu Xietong Pharmaceutical Bio-engineering Co., Ltd., Nanjing, China (product number: XT93M).

#### 2.5.2. Histopathological Examination

To assess the efficacy of *P. scabiosaefolia* in treating UC, observations of histopathological changes in the colon of the mice were conducted, along with the measurement of biochemical markers in the serum. The colon specimens were collected, rinsed with PBS, and fixed in 10% formalin. To observe the cellular structures, the sliced sections were stained by hematoxylin and eosin (HE) staining kit and Periodic acid Schiff and Alcian blue (AB-PAS, Beijing Solarbio Science & Technology Co., Ltd., Beijing, China) staining kit, respectively.

#### 2.5.3. ELISA Assay

The levels of interleukin-6 (IL-6), lipopolysaccharide (LPS), D-Lactate, and tumor necrosis factor-alpha (TNF-α, Shanghai Hualan Biotechnology Co., Ltd., Shanghai, China) in the mouse serum were measured by ELISA kits.

#### 2.5.4. Quantitative Real-Time PCR Analysis

RNA was extracted from mouse colon tissue using Trizol reagent (Thermo Fisher, Shanghai, China), and cDNA was synthesized by reverse transcription following the instructions of the All-in-One 5 × RT MasterMix kit. Gene mRNA levels were analyzed using a real-time fluorescence quantitative PCR machine (Applied Biosystems, Inc., Waltham, MA, USA) according to the BlasTaq™ 2 × qPCR MasterMix kit protocol (Abmgood, Shanghai, China). The primer sequences are in Table S1.

#### 2.5.5. High-Throughput Sequencing of Gut Microbiota

The colon contents from each group of mice were collected after dissection for DNA extraction. A gut sequencing analysis was performed by Shanghai Meiji Biotechnology Co., Ltd. (Shanghai, China).

### 2.6. Statistical Analysis

All the statistical analyses were conducted using GraphPad Prism V8.0. Data were expressed as mean ± standard deviation (SD), with a *p*-value < 0.05 considered statistically significant.

## 3. Results

### 3.1. Identification of P. scabiosaefolia Components

**Figure 1:**
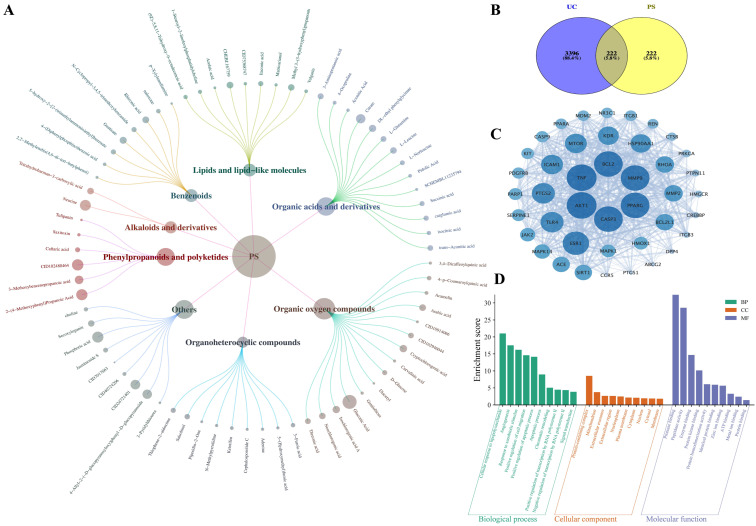
Ingredient determination and network pharmacology prediction results of *P. scabiosaefolia*. (**A**) Composition and classification of *P. scabiosaefolia*. (**B**) Venn diagram. (**C**) Interaction network of PS-UC key targets. (**D**) GO enrichment analysis (top 10).

Chemical components were identified in the extract of the medicinal and edible compound, including organic oxygen compounds, organic acids and derivatives, phenylpropanoids and polyketides, benzenoids, lipids and lipid-like molecules, alkaloids and derivatives, organoheterocyclic compounds, and others. After comparing the database with the standard products, removing duplicates, and the relative peak area ratio > 0.5%, *P. scabiosaefolia* identified a total of 72 compounds, detailly, 15 organic oxygen compounds, 14 organic acids and derivatives, 6 phenylpropanoids and polyketides, 8 benzenoids, 9 lipids and lipid-like molecules, 2 alkaloids and derivatives, 9 organoheterocyclic compounds, and 9 others (Figure 1A). Among them, the one with the highest relative content is gluconic acid (6.57%), while the total relative content of organic oxygen compounds is the highest at 17.13% (Table S2 and Figure S1). Organic oxygen compounds, such as gluconic acid, are known for their roles in metabolic processes and their potential to influence gut health [16]. Organic acids and their derivatives may contribute to the regulation of gut pH and the maintenance of a favorable environment for beneficial microbiota [17]. Moreover, compounds such as phenylpropanoids and polyketides possess anti-inflammatory and antioxidant properties, which are likely to play a significant role in reducing inflammation and oxidative stress in UC. The high content of lipids and lipid-like molecules further suggests the potential role of *P. scabiosaefolia* in regulating cellular membrane integrity, which is crucial for maintaining the intestinal barrier function. This could be particularly relevant in the context of UC, where compromised intestinal barrier integrity contributes to disease progression.

### 3.2. Network Pharmacology Analysis

Through screening in the SwissTargetPrediction database, a total of 1395 target points related to *P. scabiosaefolia* were identified. After selecting those with a probability value > 0.1 and removing duplicates, 659 target proteins associated with *P. scabiosaefolia* were obtained. Keywords such as “UC” and “ulcerative colitis” were searched in the OMIM and Genecards databases, and after applying the median method and removing duplicates, 3396 target points related to UC were retrieved. A Venn diagram was used to illustrate the overlapping targets between *P. scabiosaefolia* and UC, revealing 222 intersecting target points (Figure 1B).

These 222 intersecting targets were further analyzed for PPI data using the String database. To identify the core targets of *P. scabiosaefolia* in treating UC, a core target screening was conducted based on degree, betweenness, and closeness centrality values greater than the median of all the nodes (degree centrality > 28.15, betweenness centrality > 245.32, and closeness centrality > 0.0022). This analysis led to the creation of a network diagram containing 42 nodes and 563 connections (Figure 1C). The network reveals that *P. scabiosaefolia* interacts with several key proteins, including TNF, AKT1, CASP3, BCL2, and MMP9 (top five degree centrality), which play pivotal roles in inflammation, immune response, and cell survival. In addition, the compound–disease–target network was constructed to further identify the key components of PS in the treatment of UC. The top 15 key compounds of *P. scabiosaefolia* were selected based on the degree centrality ranking (Table 1). Among them, 4-Oxo-4-[(3-oxo-2-decanyl)amino]butanoic acid, (9Z)-5,8,11-Trihydroxy-9-octadecenoic acid, Vulgarin, 4-(Diphenylphosphino)benzoic acid, and 1-[(4-Bromo-3,5-dimethyl-1H-pyrazol-1-yl)methyl]-3,5-dimethyl-1H-pyrazol-4-amine are potential active components of *P. scabiosaefolia* in the treatment of UC.

Figure 1D shows the enrichment scores for the biological processes, cellular components, and molecular functions associated with the target proteins. Notably, the biological processes related to immune response, inflammation, and cell signaling pathways are highly enriched. These findings support the hypothesis that *P. scabiosaefolia* influences critical pathways involved in immune modulation and inflammation in UC. The enrichment of cellular components related to membranes and intracellular signaling further suggests that *P. scabiosaefolia* could affect cell function and communication within the intestinal epithelium.

### 3.3. Molecular Docking Analysis

**Figure 2:**
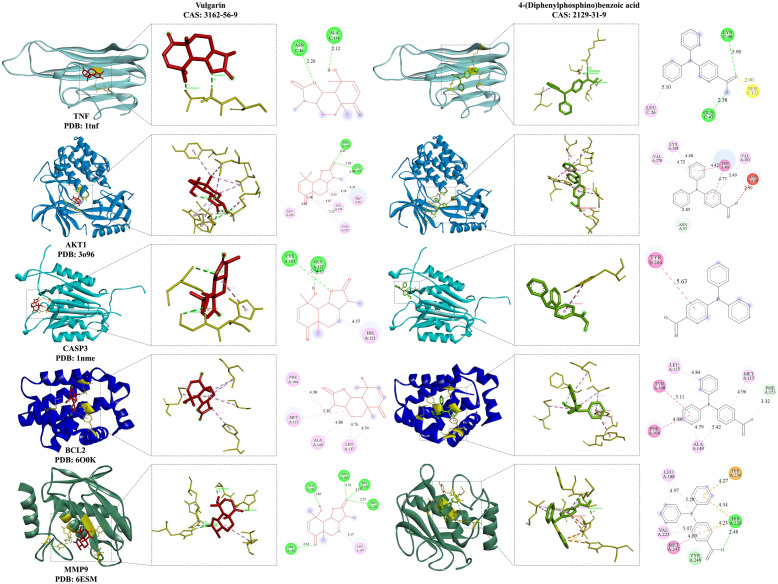
The magnitude of the binding free energy for molecular docking.

To identify the essential signaling molecules of *P. scabiosaefolia* in UC and examine the interactions between its active compounds and core targets, a molecular docking analysis was performed. The top five active compounds based on degree centrality were docked with five core targets, and the minimum binding free energy was calculated using the AutoDock software 2.7.11 (Table S3). In this study, complexes with lower binding free energy typically exhibit stronger binding affinity, correlating with higher activity. The minimum binding free energy between *P. scabiosaefolia* and UC targets was found to be −9.2 kcal/mol, while the remaining binding free energies were all below −4.6 kcal/mol. These results suggest that the core components of *P. scabiosaefolia* have a high affinity for the core targets, particularly Vulgarin and AKT1. Two active compounds—Vulgarin and 4-(Diphenylphosphino)benzoic acid—with binding free energy lower than −7 kcal/mol to five targets, were selected for visualization (Figure 2). In conclusion, both the minimum binding free energy and the visualization results support the effectiveness of the active components, with Vulgarin showing stronger binding affinity and higher activity.

### 3.4. P. scabiosaefolia Alleviated the Symptoms of UC Mice

**Figure 3:**
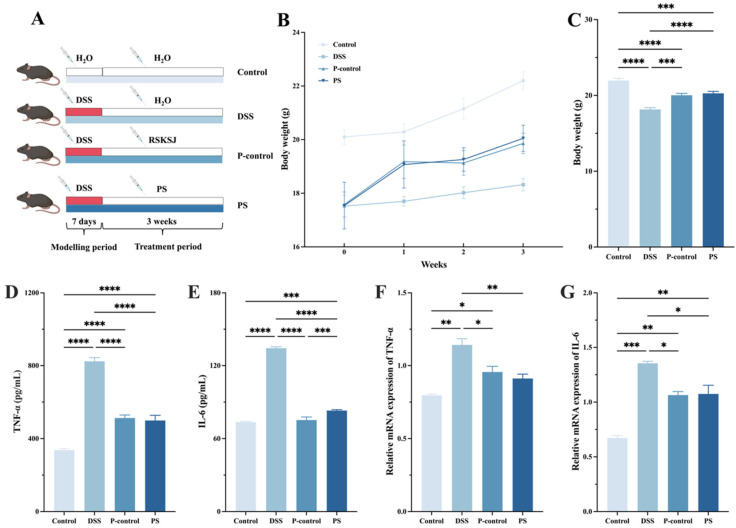
(**A**) Experimental flowchart. (**B**) Weight change. (**C**) Third-week body weight of each group. (**D**) TNF-α. (**E**) IL-6. (**F**) Relative mRNA expression of TNF-α and (**G**) IL-6. Data are presented as the mean ± SD (*n* = 7). * *p* < 0.05, ** *p* < 0.01, *** *p* < 0.001, and **** *p* < 0.0001.

The DSS-induced UC mice showed reduced weight gain compared to the control group. However, *P. scabiosaefolia* treatment facilitated body weight recovery. After three weeks, the PS group reached 20.05 ± 0.49 g, significantly surpassing the DSS model group (18.32 ± 0.23 g). Pro-inflammatory cytokines, especially TNF-α and IL-6, are key contributors to UC development [18]. As shown in Figure 3D,E, DSS administration led to a significant increase in TNF-α and IL-6 levels compared to the control group (*p* < 0.05). However, after intervention with RSKSJ and *P. scabiosaefolia*, the levels of these pro-inflammatory cytokines were significantly reduced in the UC mice. To further investigate the anti-inflammatory properties of *P. scabiosaefolia* in the UC mice, qRT-PCR was employed to measure the expression levels of pro-inflammatory cytokines, specifically TNF-α and IL-6. As shown in Figure 3F,G, the DSS treatment markedly increased the expression of TNF-α and IL-6 in the UC mice. Following the intervention, a significant reduction in TNF-α and IL-6 expression was observed in the PS group, with TNF-α levels reaching those similar to the control group (*p* > 0.05). These results suggest that *P. scabiosaefolia* intervention effectively alleviates inflammation and promotes weight recovery in the DSS-induced UC mice, highlighting its potential as a therapeutic agent for managing UC through modulation of pro-inflammatory cytokines.

### 3.5. P. scabiosaefolia Modulates the Intestinal Barrier in the Colon of UC Mice

**Figure 4:**
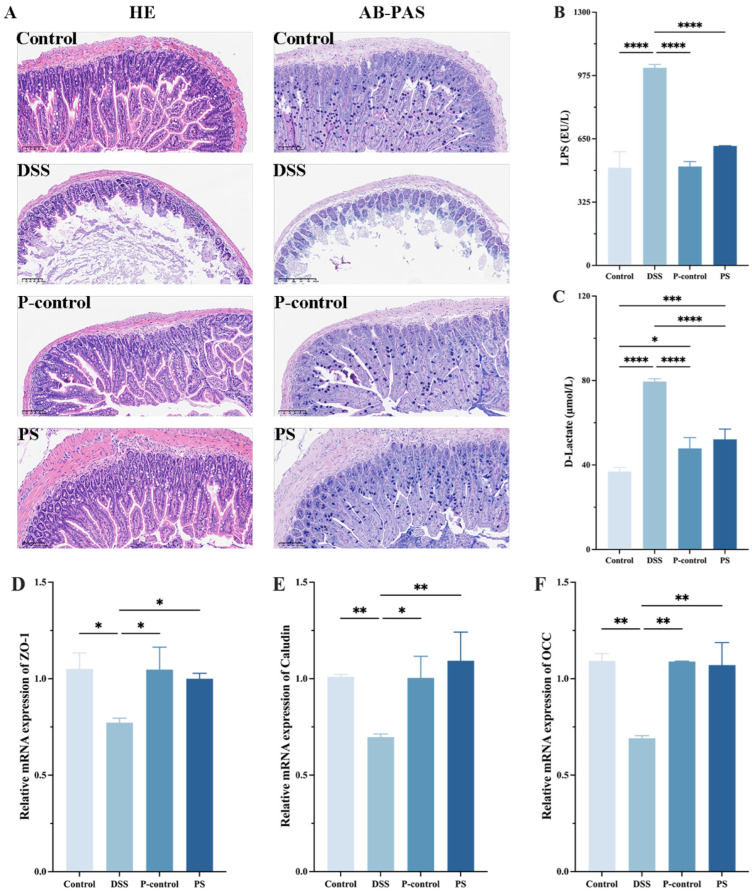
(**A**) HE staining and AB-PAS staining. (**B**) LPS. (**C**) D-Lactate. (**D**) Relative mRNA expression of ZO-1, (**E**) Claudin, and (**F**) OCC. Data are presented as the mean ± SD (*n* = 7). * *p* < 0.05, ** *p* < 0.01, *** *p* < 0.001, and **** *p* < 0.0001.

To visually evaluate the therapeutic impact of *P. scabiosaefolia* on DSS-induced ulcerative colitis, a histopathological examination of the mouse colon tissues was performed. The HE staining results revealed that compared to the control group, the DSS group exhibited significantly disordered colon glands, a loss of goblet cells, disrupted intestinal villi, and marked inflammatory infiltration. However, following *P. scabiosaefolia* intervention, the colon glands in the PS group were more orderly arranged, goblet cells were restored, intestinal villi and crypts were clearer, and inflammatory infiltration was notably reduced. AB-PAS staining further confirmed that the number of goblet cells in the DSS group was significantly decreased. After the *P. scabiosaefolia* treatment, the number of goblet cells was increased (Figure 4A). To evaluate the protective effects of *P. scabiosaefolia* on the intestinal barrier integrity in the UC mice, the serum levels of LPS and D-Lactic acid were measured. Additionally, RT-qPCR was used to assess the expression levels of intestinal tight junction proteins, including ZO-1, Claudin, and OCC. The results showed that compared to the control group, the serum levels of LPS (1015.05 ± 16.99 EU/L) and D-lactic acid (79.51 ± 1.31 μmol/L) were significantly elevated in the DSS-induced UC mice (*p* < 0.05). After 3 weeks of PS administration, the serum levels of LPS (613.89 ± 0.82 EU/L) and D-lactic acid (52.13 ± 4.89 μmol/L) were significantly reduced (*p* < 0.05) (Figure 4B,C). DSS stimulation led to altered expression levels of ZO-1, Claudin, and OCC in the colon of UC mice. After *P. scabiosaefolia* intervention, the PS group exhibited tight junction protein expression levels comparable to those of the control group (Figure 4D–F) (*p* > 0.05). These data suggest that *P. scabiosaefolia* effectively protects the intestinal barrier integrity in UC mice.

### 3.6. P. scabiosaefolia Modulates the Gut Microbiota in UC Mice

**Figure 5:**
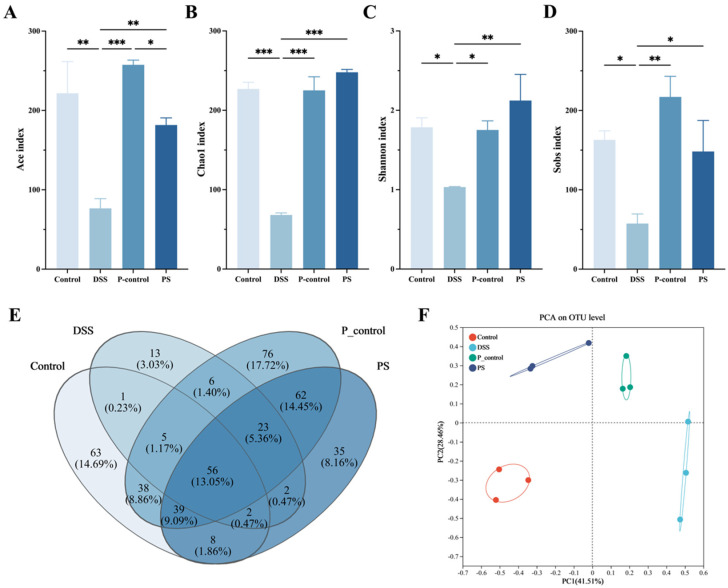
(**A**) Ace index. (**B**) Chao1 index. (**C**) Shannon index. (**D**) Sobs index. (**E**) Venn diagram. (**F**) PCA on the OUT level. Data are presented as the mean ± SD (*n* = 3). * *p* < 0.05, ** *p* < 0.01, and *** *p* < 0.001.

Figure 5 demonstrates that *P. scabiosaefolia* effectively modulates the gut microbiota to alleviate DSS-induced UC in the mice. An analysis of alpha diversity indices (Ace, Chao1, Shannon, and Sobs indices) (Figure 3A–D) revealed that the *P. scabiosaefolia* treatment significantly enhanced microbial diversity and richness compared to the DSS group (*p* < 0.05). Specifically, the PS group exhibited higher values across all the indices, indicating that PS aids in restoring the gut microbiota disrupted by DSS, promoting both greater diversity and balance. The Venn diagram further highlighted that *P. scabiosaefolia* treatment increased the overlap of operational taxonomic units (OTUs), suggesting a recovery of microbial communities affected by DSS (Figure 3E). A PCA analysis of the intestinal flora levels revealed a strong similarity between the PS group and the control group (Figure 3F). Collectively, these results indicate that *P. scabiosaefolia* administration restores microbial diversity and richness.

**Figure 6:**
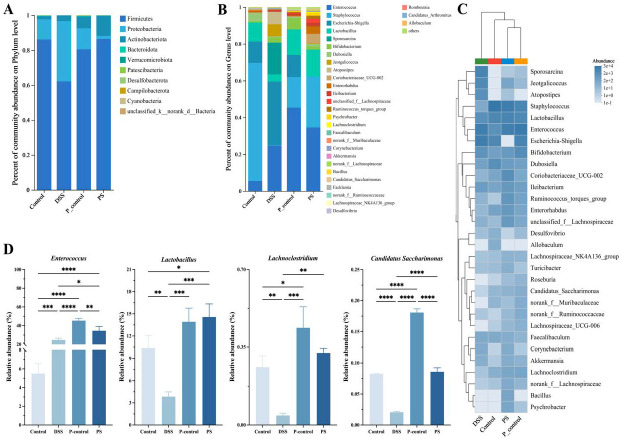
(**A**) Phylum. (**B**) Genus. (**C**) Differences at the genus level. (**D**) Heatmap and cluster analysis on genus level. Data are presented as the mean ± SD (*n* = 3). * *p* < 0.05, ** *p* < 0.01, *** *p* < 0.001, and **** *p* < 0.0001.

At the phylum level (Figure 6A), the microbiota composition of the PS group closely resembles that of the control group, with a significant restoration of microbial diversity compared to the DSS group. Specifically, the PS group effectively increased the level of *Firmicutes* and decreased the level of *Proteobacteria*. The *P. scabiosaefolia* intervention promoted a more balanced distribution. At the genus level (Figure 6B), the *P. scabiosaefolia* treatment significantly altered the microbial composition, increasing the relative abundance of beneficial genera like *Lactobacillus*, *Bifidobacterium*, and *Enterococcus*, all of which are known to have protective effects on gut health [19]. In contrast, the DSS group displayed higher levels of potentially pathogenic genera such as *Escherichia-Shigella* and *Sporosarcina*, which are often associated with inflammation and gut dysbiosis. The heatmap further highlights these differences in microbial composition, with the PS group showing significantly higher abundance of beneficial bacteria (such as *Lactobacillus* and *Bifidobacterium*), while the DSS group exhibited higher levels of *Escherichia-Shigella* and *Sporosarcina*. A cluster analysis of the intestinal flora levels indicated a strong similarity between the PS group and the control group (Figure 4C). The PS group exhibited a clear shift towards a healthier microbiota profile, with increased beneficial bacteria and decreased harmful genera.

Figure 4D focuses on the relative abundance of specific genera, namely *Enterococcus*, *Lactobacillus*, *Lachnoclostridium*, and *Candida Saccharimonas*. The *P. scabiosaefolia* treatment led to a significant increase in the abundance of *Enterococcus* and *Lactobacillus*, both of which are beneficial to gut health and may help improve intestinal barrier function. Additionally, *Lachnoclostridium* showed a significant increase in the PS group, which is consistent with its role in maintaining gut health, while *Candida Saccharimonas* was notably reduced. The reduction in *Candida Saccharimonas* is particularly noteworthy, as this genus is associated with gut inflammation and microbial imbalance. In summary, *P. scabiosaefolia* treatment effectively modulates the gut microbiota in UC mice by promoting beneficial bacterial populations and reducing harmful ones.

## 4. Discussion

Compared with the side effects of Western medicine or traditional Chinese medicine, the intervention of medicinal food homology decoction for UC is an important approach. In this study, we identified a diverse range of active compounds in *P. scabiosaefolia*, and demonstrated that its extract improves DSS-induced UC by modulating the gut microbiota. Moreover, *P. scabiosaefolia* significantly reduced the levels of IL-6 and TNF-α, along with their mRNA expression, highlighting its ability to effectively inhibit the inflammatory response. Additionally, we confirmed that *P. scabiosaefolia* promotes the repair of the intestinal epithelial mucosal barrier damaged by DSS by upregulating the expression of tight junction proteins, including ZO-1, OCC, and Claudin.

*P. scabiosaefolia* used in this study contains a variety of bioactive substances, including organic oxygen compounds, organic acids and derivatives, phenylpropanoids and polyketides, benzenoids, alkaloids, and derivatives (Figure 1A). Currently, there are few reports on the use of *P. scabiosaefolia* for the treatment of UC. We employed network pharmacology and molecular docking to explore the therapeutic potential of *P. scabiosaefolia* in treating DSS-induced UC. The UHPLC-OE-MS/MS allowed us to identify a diverse range of bioactive compounds, including vulgarin and 4-(Diphenylphosphino)benzoic acid, which have shown potential in modulating key biological pathways associated with inflammation and immune responses. Meanwhile, the molecular docking results revealed that vulgarin and 4-(Diphenylphosphino)benzoic acid bind effectively to targets such as TNF, AKT1, CASP3, BCL2, and MMP9, all of which are critical in the inflammatory cascade underlying UC. Specifically, TNF is a classic pro-inflammatory cytokine, indirectly activating endothelial cells through inflammation in target cells. AKT1 regulates inflammation and immune responses, while CASP3 and BCL2 play critical roles in cell apoptosis and survival, central to the pathophysiology of UC [20]. Furthermore, MMP9, a matrix metalloproteinase, is implicated in tissue remodeling and inflammation [2]. Overall, *P. scabiosaefolia* may exert its therapeutic effects by modulating multiple biological processes such as inflammation, cell survival, and tissue repair, providing a comprehensive mechanism for its beneficial impact on UC.

In the DSS-induced colitis mouse model, we observed that *P. scabiosaefolia* effectively suppressed the secretion of TNF-α and IL-6 and downregulated their mRNA transcription levels. This suggests that *P. scabiosaefolia* may modulate the immune response by interacting with signaling pathways involved in immune cell activation and the subsequent release of pro-inflammatory cytokines. Due to the complexity of the compounds in the *P. scabiosaefolia* extract, pinpointing their direct targets remains challenging. Based on the current research, we hypothesize that the therapeutic effects of *P. scabiosaefolia* in treating UC could be attributed to the synergistic action of multiple targets. Among the core components identified, several have been reported to exhibit anti-inflammatory properties. For instance, Vulgarin has been shown to reduce IL-6 secretion [21], and 3,4-Dicaffeoylquinic acid has been demonstrated to attenuate the production of NO and TNF-α in RAW 264.7 macrophage cells [22].

In addition to these direct effects, the modulation of the gut microbiota structure by *P. scabiosaefolia* is also one of the mechanisms through which it treats UC. During the long-term evolution process, intestinal flora continues to interact with the host to regulate the intestinal environmental homeostasis, including regulating intestinal immune function, and protecting intestinal epithelial cells from damage. The findings indicate that *P. scabiosaefolia* significantly contributes to maintaining the integrity of the intestinal barrier, as demonstrated by the increased expression of tight junction proteins ZO-1, OCC, and Claudin (Figure 4D–F). This suggests that *P. scabiosaefolia* may facilitate the restoration of the intestinal epithelial mucosal barrier, a crucial factor in preserving gut homeostasis and mitigating inflammatory progression in UC. It is worth noting that in the PS group, the relative abundance of beneficial bacteria (*Lactobacillus* and *Lachnoclostridium*) significantly increased (*p* < 0.05), while harmful bacteria (*Enterococcus* and *Candidatus Saccharimonas*) were significantly reduced (*p* < 0.05) (Figure 5D). Studies have shown that *Lachnoclostridium* can significantly reduce DSS-induced colonic mucosal injury and inflammation levels, and *Lactobacillus* also exhibits protective effects in DSS-induced colitis models in mice [23]. These findings align with an increasing body of evidence, which suggests that the modulation of the gut microbiota is crucial for resolving intestinal inflammation and improving the overall health of the intestinal barrier. More importantly, some active compounds, such as gluconic acid, may play a key role in this process. Research has shown that by feeding gluconic acid, which may be mediated by butyrate, an increase in mRNA anti-inflammatory cytokines in the distal small intestine mucosa and survival signaling levels of the distal small intestine mucosa were found [16]. These findings strongly suggest that the therapeutic effects of PS in treating ulcerative colitis may result from the synergistic action of multiple pathways.

## 5. Conclusions

In conclusion, *P. scabiosaefolia* exhibits strong immune-regulatory functions, specifically by reducing the secretion of pro-inflammatory cytokines. Additionally, *P. scabiosaefolia* can restore intestinal barrier function and modulate the composition and levels of the gut microbiota, thereby improving UC. Our results indicate that PS could serve as a promising therapeutic option for UC treatment.

## Figures and Tables

**Table 1:** Key compounds of *P. scabiosaefolia* (top 15 by degree centrality).

Key Compounds	Dgree Centrality
4-Oxo-4-[(3-oxo-2-decanyl)amino]butanoic acid	18
(9Z)-5,8,11-Trihydroxy-9-octadecenoic acid	16
Vulgarin	14
4-(Diphenylphosphino)benzoic acid	13
1-[(4-Bromo-3,5-dimethyl-1H-pyrazol-1-yl)methyl]-3,5-dimethyl-1H-pyrazol-4-amine	7
Ketotifen	6
Methyl 3-(3-hydroxyphenyl)propanoate	6
2-(4-Methoxyphenyl)Propanoic Acid	5
3-Methoxybenzenepropanoic acid	4
5-hydroxy-2-[2-(trimethylammonio)ethyl]benzoate	4
Pidolic Acid	4
1-methyl-1,2,3,4-tetrahydro-beta-carboline-3-carboxylic acid	3
Azelaic acid	3
N-Cyclopropyl-3,4,5-trimethoxybenzamide	3
4-Oxoproline	2

## Data Availability

The original contributions presented in this study are included in the article/Supplementary Materials. Further inquiries can be directed to the corresponding author.

## Supplementary Materials

The following supporting information can be downloaded at https://www.mdpi.com/article/10.3390/foods14071145/s1, Figure S1: UHPLC-OE-MS/MS chromatogram of PS (A) NEG (B) POS; Table S1: Primer Sequences for RT-qPCR analysis; Table S2: Concentration (%) of phytochemical compounds in PS identified by UHPLC-OE-MS/MS; Table S3: Molecular docking results of key substances and core targets.
