# Supplementary material for: Patrinia scabiosaefolia L. Modulates the Intestinal Microecology to Treat DSS-Induced Ulcerative Colitis: UHPLC-OE-MS/MS, Network Pharmacology, and Experimental Validation
Source: Foods. 2025 Mar 25;14(7):1145. doi: 10.3390/foods14071145 (PMC11988699; doi:10.3390/foods14071145)
Supplement: Supplementary file 1 [file foods-14-01145-s001.zip › foods-3510396-supplementary.pdf]

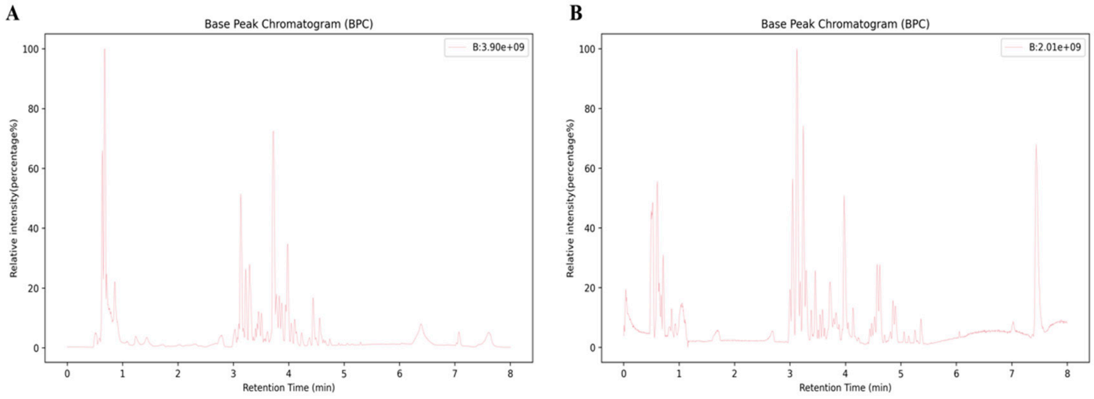

Figure S1. UHPLC-OE-MS/MS chromatogram of PS (A) NEG (B) POS.

Table S1. Primer Sequences for RT-qPCR Analysis.

Name	Primer sequence
IL-6	Forward primer: 5′-AACTGGAAAGGAGACAGCAAG-3′<br>Reverse primer: 5′-AAGTAGGGAAGGCCGTGG-3′
TNF-α	Forward primer: 5′-AAGGAGAACCAAGTGGTGAG-3′<br>Reverse primer: 5′-GAGGCCATTGGCCAGGG-3′
ZO-1	Forward primer: 5′-CCGAGGACTACAGAGACCA-3′<br>Reverse primer: 5′-CCAGTGAGGACGACAAAGAA-3′
Caludin	Forward primer: 5′-GAGAGAGGAGACAGGAAGG-3′<br>Reverse primer: 5′-TCAAGATGACAGAGGGAGG-3′
OCC	Forward primer: 5′-TGGAGAGGGTGAGAGGAAG-3′<br>Reverse primer: 5′-AGAGGACGAGTGGGAGGAAG-3′

Table S2. Concentration (%) of phytochemical compounds in PS identified by UHPLC-OE-MS/MS.

No.	Name	Formula	M/Z (Da)	RT (s)	Adduct	Concentration (%)
1	Gluconic Acid	$C_6H_{12}O_7$	195.05	0.632	neg	6.57
2	7-hydroxy-5-methyl-4-[(2S,3R,4S,5S,6R)-3,4,5-trihydroxy-6-(hydroxymethyl)oxan-2-yl]oxychromen-2-one	$C_{16}H_{18}O_9$	353.09	3.297	neg	4.15
3	2-(4-Methoxyphenyl)Propanoic Acid	$C_{10}H_{12}O_3$	181.09	3.982	pos	3.99
4	Phosphoric acid	H_3O_4P	96.96	0.828	neg	3.93
5	Neurine	$C_5H_{13}NO$	104.11	0.611	pos	3.72
6	(3Z)-4-{2-[2-(3,4-Dihydroxyphenyl)ethoxy]-2-oxoethyl}-3-ethylidene-2-(beta-D-glucopyranosyloxy)-3,4-dihydro-2H-pyran-5-carboxylic acid	$C_{24}H_{30}O_{13}$	526.49	3.222	neg	2.91
7	4-Allyl-2-(-D-glucopyranosyloxy)phenyl -D-glucopyranoside	$C_{21}H_{30}O_{12}$	473.17	3.954	neg	2.74
8	Citrate	$C_6H_8O_7$	191.02	0.774	neg	2.70
9	3-Methoxybenzenepropanoic acid	$C_{10}H_{12}O_3$	181.09	3.723	pos	2.19
10	Cryptochlorogenic acid	$C_{16}H_{18}O_9$	355.10	3.295	pos	1.60
11	Isochlorogenic acid A	$C_{25}H_{24}O_{12}$	515.12	3.792	neg	1.54
12	DL-ethyl phenylglycinate	$C_{10}H_{13}NO_2$	179.22	3.98	pos	1.50

13	Gentisate	$C_7H_6O_4$	153.02	2.767	neg	1.40
14	Rhizonic acid	$C_{10}H_{12}O_4$	197.08	3.47	pos	1.29
15	4-Oxo-4-[(3-oxo-2-decanyl)amino]butanoic acid	$C_{14}H_{25}NO_4$	294.17	3.267	pos	1.29
16	Arabic acid	$C_5H_{10}O_6$	165.04	0.636	neg	1.28
17	1-methyl-1,2,3,4-tetrahydro-beta-carboline-3-carboxylic acid	$C_{13}H_{14}N_2O_2$	188.07	3.009	pos	1.22
18	L-Leucine	$C_6H_{13}NO_2$	132.10	1.048	pos	1.20
19	Neochlorogenic acid	$C_{16}H_{18}O_9$	353.09	3.016	neg	1.17
20	Methyl 3-(3-hydroxyphenyl)propanoate	$C_{10}H_{12}O_3$	181.09	3.828	pos	1.17
21	Secoxyloganin	$C_{23}H_{34}O_{16}$	403.12	3.454	neg	1.15
22	3-Aminopentanoic acid	$C_5H_{11}NO_2$	118.09	0.641	pos	1.00
23	L-Glutamine	$C_5H_{10}N_2O_3$	147.08	0.623	pos	0.92
24	Threonic acid	$C_4H_8O_5$	135.03	0.66	neg	0.91
25	trans-Aconitic acid	$C_6H_6O_6$	173.01	1.09	neg	0.88
26	(1S,4aR,7aR)-4a-hydroxy-7-methylidene-1-[(2S,3R,4S,5S,6R)-3,4,5-trihydroxy-6-(hydroxymethyl)oxan-2-yl]oxy-1,5,6,7a-tetrahydrocyclopenta[c]pyran-4-carbaldehyde	$C_{16}H_{22}O_9$	359.13	3.456	pos	0.88
27	Salsolinol	$C_{10}H_{13}NO_2$	180.10	0.842	pos	0.79
28	carglumic acid	$C_6H_{10}N_2O_5$	190.15	0.868	pos	0.74
29	N-Cyclopropyl-3,4,5-trimethoxybenzamide	$C_{13}H_{17}NO_4$	252.12	1.04	pos	0.72
30	Itaconic acid	$C_5H_6O_4$	129.02	1.092	neg	0.72
31	2-Thiophenecarboxaldehyde Oxime	C_5H_5NOS	128.02	0.52	pos	0.72
32	5-hydroxy-2-[2-(trimethylammonio)ethyl]benzoate	$C_{12}H_{17}NO_3$	224.13	0.711	pos	0.71
33	Succinic acid	$C_4H_6O_4$	117.02	1.083	neg	0.68
34	Caftaric acid	$C_{13}H_{12}O_9$	312.23	3.475	neg	0.68
35	D-Glucose	$C_6H_{12}O_6$	179.06	0.627	neg	0.68
36	Jasminoside Q	$C_{22}H_{36}O_{12}$	491.21	4.136	neg	0.64
37	L-Norleucine	$C_6H_{13}NO_2$	132.10	0.94	pos	0.64
38	4-p-Coumaroylquinic acid	$C_{16}H_{18}O_8$	337.09	3.493	neg	0.56
39	naloxone	$C_{19}H_{21}NO_4$	328.15	3.387	pos	0.56
40	3-Furoic acid	$C_5H_4O_3$	111.01	0.858	neg	0.53
41	Choline	$C_5H_{14}NO$	104.11	0.774	pos	0.51
42	isocitric acid	$C_6H_8O_7$	173.01	0.853	neg	0.50
43	(9Z)-5,8,11-Trihydroxy-9-octadecenoic acid	$C_{18}H_{34}O_5$	329.23	4.485	neg	0.50
44	1-Stearoyl-2-linoleoylphosphatidylcholine	$C_{44}H_{84}NO_8P$	786.60	5.761	pos	0.46
45	(1R,9S)-11-(Cyclohexylcarbonyl)-5-[4-(methylsulfanyl)phenyl]-7,11-diazatricyclo[7.3.1.02,7]trideca-2,4-dien-6-one	$C_{25}H_{30}N_2O_2S$	457.17	3.663	neg	0.44
46	4-Oxoproline	$C_5H_7NO_3$	128.04	0.867	neg	0.42
47	Acamelin	$C_{10}H_8O_4$	193.05	3.886	pos	0.41
48	Ketotifen	$C_{19}H_{19}NOS$	310.13	1.372	pos	0.40
49	Pidolic Acid	$C_5H_7NO_3$	128.04	0.949	neg	0.39
50	Vulgarin	$C_{15}H_{20}O_4$	265.14	4.528	pos	0.39
51	4-(Diphenylphosphino)benzoic acid	$C_{19}H_{15}O_2P$	305.07	3.573	neg	0.37
52	6-O-(2-Methylbutanoyl)-alpha-D-glucopyranosyl alpha-D-glucopyranoside	$C_{17}H_{30}O_{12}$	426.40	3.379	neg	0.36
53	Aconitic Acid	$C_6H_6O_6$	173.01	0.916	neg	0.36
54	Saxitoxin	$C_{10}H_{17}N_7O_4$	299.29	0.7	pos	0.34
55	Gentiobiose	$C_{12}H_{22}O_{11}$	341.11	0.643	neg	0.34
56	Matricarianol	$C_{10}H_{10}O$	145.06	0.613	neg	0.33

57	Adenine	$C_5H_5N_5$	136.06	0.69	pos	0.33
58	N-Methylpyrrolidine	$C_5H_{11}N$	86.10	1.224	pos	0.33
59	5-(Hydroxymethyl)furoic acid	$C_6H_6O_4$	143.03	1.05	pos	0.33
60	3,4-Dicaffeoylquinic acid	$C_{25}H_{24}O_{12}$	516.40	3.795	pos	0.32
61	Curvulinic acid	$C_{10}H_{10}O_5$	211.06	3.224	pos	0.31
62	2,2'-Methylenebis(4,6-di-tert-butylphenol)	$C_{29}H_{44}O_2$	423.33	7.104	neg	0.31
63	Cephalosporolide C	$C_{10}H_{16}O_5$	217.11	1.12	pos	0.31
64	(2S)-2-amino-5-hydroxy-5-oxopentanoate;hydron	$C_5H_9NO_4$	146.05	0.62	neg	0.30
65	5-(beta-D-Glucopyranosyloxy)-2-hydroxybenzoic acid	$C_{13}H_{16}O_9$	315.07	2.139	neg	0.30
66	Azelaic acid	$C_9H_{16}O_4$	187.10	3.973	neg	0.29
67	Piperidin-2-One	C_5H_9NO	100.08	7.604	pos	0.29
68	3-Pyridylthiourea	$C_6H_7N_3S$	154.04	3.056	pos	0.28
69	Diacetyl	$C_4H_6O_2$	85.03	1.094	neg	0.27
70	p-Xylylenediamine	$C_8H_{12}N_2$	136.19	1.697	pos	0.27
71	Tulipanin	$C_{27}H_{31}O_{16}$	609.15	3.578	neg	0.27
72	1-[(4-Bromo-3,5-dimethyl-1H-pyrazol-1-yl)methyl]-3,5-dimethyl-1H-pyrazol-4-amine	$C_{11}H_{16}BrN_5$	298.18	7.281	pos	0.25

Table S3. Molecular docking results of key substances and core targets.

**Compounds**	**Binding Free Energy (kcal/mol)**				
	**TNF**	**AKT1**	**CASP3**	**BCL2**	**MMP9**
4-Oxo-4-[(3-oxo-2-decanyl)amino]butanoic acid	-4.6	-6.7	-5	-6.2	-6.9
(9Z)-5,8,11-Trihydroxy-9-octadecenoic acid	-4.6	-6.6	-5.6	-6	-7.2
Vulgarin	-6.4	-9.2	-6.3	-7.2	-7.3
4-(Diphenylphosphino)benzoic acid	-6.3	-8.2	-6.5	-7.7	-8.6
1-[(4-Bromo-3,5-dimethyl-1H-pyrazol-1-yl)methyl]-3,5-dimethyl-1H-pyrazol-4-amine	-5	-7.1	-5.5	-6.2	-7.4
